# Plant Density Effect on Grain Number and Weight of Two Winter Wheat Cultivars at Different Spikelet and Grain Positions

**DOI:** 10.1371/journal.pone.0155351

**Published:** 2016-05-12

**Authors:** Yong Li, Zhengyong Cui, Yingli Ni, Mengjing Zheng, Dongqing Yang, Min Jin, Jin Chen, Zhenlin Wang, Yanping Yin

**Affiliations:** 1 National Key Laboratory of Crop Biology, Agronomy College of Shandong Agricultural University, Tai’an, Shandong, P. R. China; 2 Agricultural Bureau of Rencheng District, Jining, Shandong, P. R. China; 3 Agricultural Bureau of Zhangqiu District, Jinan, Shandong, P. R. China; Institute of Genetics and Developmental Biology, Chinese Academy of Sciences, CHINA

## Abstract

In winter wheat, grain development is asynchronous. The grain number and grain weight vary significantly at different spikelet and grain positions among wheat cultivars grown at different plant densities. In this study, two winter wheat (*Triticum aestivum* L.) cultivars, ‘Wennong6’ and ‘Jimai20’, were grown under four different plant densities for two seasons, in order to study the effect of plant density on the grain number and grain weight at different spikelet and grain positions. The results showed that the effects of spikelet and grain positions on grain weight varied with the grain number of spikelets. In both cultivars, the single-grain weight of the basal and middle two-grain spikelets was higher at the 2^nd^ grain position than that at the 1^st^ grain position, while the opposite occurred in the top two-grain spikelets. In the three-grain spikelets, the distribution of the single-grain weight was different between cultivars. In the four-grain spikelets of Wennong6, the single-grain weight was the highest at the 2^nd^ grain position, followed by the 1^st^, 3^rd^, and 4^th^ grain positions. Regardless of the spikelet and grain positions, the single-grain weight was the highest at the 1^st^ and 2^nd^ grain positions and the lowest at the 3^rd^ and 4^th^ grain positions. Overall, plant density affected the yield by controlling the seed-setting characteristics of the tiller spike. Therefore, wheat yield can be increased by decreasing the sterile basal and top spikelets and enhancing the grain weight at the 3^rd^ and 4^th^ grain positions, while maintaining it at the 1^st^ and 2^nd^ grain positions on the spikelet.

## Introduction

Wheat (*Triticum aestivum* L.) is the second most widely produced crop in the world [1] and the demand for food is projected to be doubled by 2050 in addition to the increasing demand for high-quality food for a healthy diet [2]. Wheat production needs to be boosted mainly through improvements in yield and agronomic practices, as the cultivated area can only marginally increase [3–5]. Therefore, greater efforts need to be made to breed new wheat varieties with higher yield potential and improve crop management in order to enhance the average farm yields.

The increase in grain number per spike contributes considerably to improve wheat grain yield potential. A wheat spike is composed of spikelets which produce reproductive structures called florets. The grain number and grain weight in wheat are influenced by both genetic and environmental factors [6]. Within a spike, they are unevenly distributed and largely different due to its unbalanced development [7–9], while they depend on the spatial position of the kernel [10]. The spatial distribution of the grain number and grain weight shows parabolic changes, and as a result, the basal grains in the central spikelets are heavier than those in the near apical and near basal spikelets [11]. Within the central spikelets, the weight at the 2^nd^ grain position is higher than that at the 1^st^, 3^rd^, and 4^th^ grain positions, when moving from the most proximal to the most distal spikelet positions [6, 11, 12]. These differences can be attributed to the poor filling of inferior spikelets, due to carbon limitations [13–15], sink capacity limitations [16], unbalanced hormone levels [17, 18], the low gene expression and activity of enzymes involved in the sucrose-to-starch conversion [19–21], and impediments to assimilate transportation [22, 23].

Plant density is an important factor that influences the growth and yield formation in wheat [24, 25]. Previous studies have focused on identifying the optimal density for wheat cultivation, but the results vary based on the experimental conditions and tested parameters [26, 27]. In wheat, the number of spikelets per spike changes under different planting densities [28]. Qu et al. [29] reported that grain yield was improved with increasing plant density as a result of the increased spikelets number. Yoshida [30] reported that dense planting maximizes the leaf area index (LAI), ensuring that plant photosynthesis meets the high yield requirements. Nakano et al. [31] also reported that dense planting might increase the grain yield, the percentage of filled spikelets, and the 1000-grain weight in forage rice (*Oryza sativa* L.). However, some studies have reported that dense planting does not necessarily increase the grain yield [32–34]. Overall, there is limited information about the effect of plant density on seed-setting traits at different spikelet and grain positions between the main stem and tiller spikes.

The objective of this study was to elucidate the effect of plant density on the grain number and grain weight at different spikelet and grain positions in winter wheat. The obtained information will help to provide a theoretical basis for the development of wheat varieties with improved yield potential.

## Materials and Methods

### Plant material and experimental design

The field experiments were carried out for two growing seasons from October 2010 to June 2011 and from October 2011 to June 2012 at the Tai’an Experimental Station of Shandong Agricultural University, Tai’an, China (36° 09’ N, 117° 09’ E; 128 m above sea level). Maize (*Zea mays* L.) was the previous crop. The soil was a sandy loam, and the 0–20-cm layer contained 12.9 g kg^-1^ total organic matter, 120 mg kg^-1^ total nitrogen (N), 87.2 mg kg^-1^ available N, 64.2 mg kg^-1^ available phosphate (PO_4_^3-^), and 109 mg kg^-1^ available potassium (K). In both seasons, fertilizer was applied before planting at a rate of 120 kg N ha^-1^ (46% N in urea), 100 kg P_2_O_5_ ha^-1^ (16% P_2_O_5_ in triple superphosphate), and 120 kg K_2_O ha^-1^ (60% K_2_O in muriate of potash). Additionally, topdressing N was applied at a rate of 120 kg N ha^-1^ during the jointing stage in the spring (GS31) [35].

The experimental arrangement was a 2 × 4 split-plot with three replicates (split plot design), in which cultivar was the primary factor and plant density was the secondary factor. We used two local winter wheat (*Triticum aestivum* L.) cultivars (‘Wennong6’, a large-spike cultivar with a relatively low number of tillers and ‘Jimai20’, a medium-spike cultivar with a relatively high number of tillers) and four plant densities (75 [D1], 225 [D2], 375 [D3], 525 plants m^-2^ [D4]). Each experimental plot consisted of 10 rows with 25 cm in-row spacing, while plant-to-plant spacing was 4.65 cm in D1, 1.50 cm D2, 0.90 cm in D3, and 0.64 cm in D4.

Seeds were sown on October 10 in 2010 and 2011 and harvested on June 16 in 2011 and June 14 in 2012. No noticeable crop damage was observed due to weeds, insects, or diseases. All agronomic practices were performed according to the precise high-yielding cultivation system as described by Yu [36].

### Plant sampling

**Fig 1:**
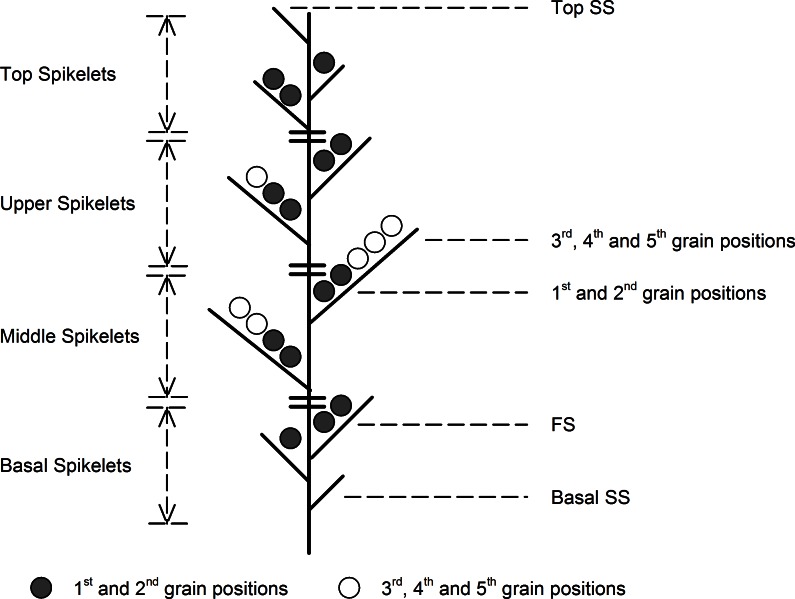
Schematic diagram indicating the specific spikelet and grain positions analyzed in detail. Fertile and sterile spikelets are classified by whether or not there are grains in the spikelets. SS, sterile spikelets (SSN, number of sterile spikelets per spike); FS, fertile spikelets (FSN, number of fertile spikelets per spike); GNS, grain number per spikelet; SGW, single-grain weight; GWS, grain weight per spikelet.

A total of 100 spikes, flowering on the same date, were labeled in two central rows of each plot, and 50 of those were removed at harvest (50 spikes × 3 replications). Each spike was divided into four parts as follows **(Fig 1)**: the basal spikelets (basal–6^th^ spikelet), middle spikelets (7^th^–15^th^ spikelet), upper spikelets (16^th^–20^th^ spikelet), and top spikelets (21^st^–terminal spikelet). The grains of each spike were numbered according to the grain position. Samples collected along 1 m of a separate central row were used to evaluate seed-setting traits at different spikelet and grain positions of the main stem and tiller spikes, while 1 m^2^ in each plot was harvested to evaluate yield.

### Statistical analysis

All traits were evaluated in triplicate. Analysis of variance in conjunction with Student–Newman–Keuls test was performed to identify significant differences between the treatments at *P* < 0.05. Statistical analysis was carried out using SPSS 18.0 (IBM Corp., Chicago, IL, USA)

## Results

### Differences in experimental factors

Table 1 presents the mean square significance between treatments and interactions for the number of spikes per plant (SP), the number of fertile spikelet per spike (FSN), the number of sterile spikelets per spike (SSN) at the top, the number of sterile spikelets per spike (SSN) at the bottom, the number of grains per spike (GN), 1,000-grain weight (TGW), spike number m^-2^ (SN), and grain yield m^-2^ (GY). Plant density and cultivar × density (C × D) interaction significantly affected SP, basal SSN, GN, TGW, SN, and GY, but not FSN or top SSN. All the traits, except for basal SSN, were significantly affected by cultivar (Table 1). Variations due to year (Yr) or Yr × C and Yr × D interactions were not significant (Table 1).

**Table 1:** Mean square significance among treatments and interactions for grain yield and its component in winter wheat.

Source of variation	SP	FSN	Top SSN	Basal SSN	GN	TGW	SN	GY
Year (Yr) ^a^	NS	NS	NS	NS	NS	NS	NS	NS
Cultivar (C)	**	*	NS	*	**	**	*	**
Plant density (D)	**	NS	NS	*	*	*	*	*
Yr × C	NS	NS	NS	NS	NS	NS	NS	NS
D × Yr	NS	NS	NS	NS	NS	NS	NS	NS
C × D	*	NS	NS	*	*	*	*	*

^a^Experiments were conducted at the Shandong Agricultural University Research Farm, Shandong Province, China, in the growing seasons 2010/2011 and 2011/2012.

SP, number of spikes per plant; FSN, number of fertile spikelets per spike; SSN, number of sterile spikelets per spike; GN, number of grains per spike; TGW, 1,000-grain weight; SN, number of spikes m^-2^; GY, grain yield m^-2^; Yr × C, year by cultivar interaction; D × Yr, plant density by year interaction; C × D, cultivar by plant density interaction. NS, not significantly different at *P* < 0.05

*, significantly different at *P* < 0.05

**, significantly different at *P* < 0.01.

### Effects of plant density on seed-setting traits of the main stem and tiller spikes

The FSN, GN, and TGW of Wennong6 were higher than those of Jimai20, while the SP and SN of Wennong6 were lower than those of Jimai20 under the same plant density conditions (Table 2). The SP, GN, and TGW of both cultivars significantly reduced with the increasing plant density (Table 2). However, SN showed different patterns of change in response to plant density between cultivars. The SN of Wennong6 increased with the increasing plant density, while that of Jimai20 was the highest in D3. Plant density had little influence on FSN; however, SSN tended to increase with the increasing plant density. The yield of both cultivars was the highest in D2 and progressively reduced in D3, D4, and D1 (Table 2). These results indicated that a moderate plant density had a positive effect on GY, while a low or high plant density could negatively affect GY.

**Table 2:** Seed-setting traits of spike and yield components in two wheat cultivars, ‘Wennong6’ and ‘Jimai20’, grown under four different plant densities.

Treatment	SP	FSN	SSN	SN	GN	TGW (g)	GY (g m^-2^)
Wennong6							
D1	4.05±0.03a	19.60±0.28a	3.35±0.02c	389±5c	56.01±0.28a	47.56±0.16a	896.6±5.6c
D2	2.59±0.02b	19.37±0.25a	3.48±0.14bc	553±5b	54.11±0.15b	47.31±0.22a	1055.8±23.1a
D3	1.46±0.01c	19.44±0.15a	3.67±0.07ab	597±4a	51.48±0.35c	44.21±0.06b	996.6±11.7b
D4	1.15±0.01d	19.97±0.10a	3.86±0.03a	614±9a	48.59±0.30d	41.84±0.34c	964.6±12.2b
Jimai20							
D1	4.70±0.18a	17.15±0.11a	3.14±0.09c	456±14c	39.41±0.28a	36.71±0.29a	719.1±7.2d
D2	3.73±0.03b	17.04±0.42a	3.60±0.05b	837±13b	35.65±0.28b	35.03±0.20a	948.5±5.9a
D3	2.48±0.02c	17.21±0.07a	3.90±0.03a	933±6a	31.52±0.06c	32.45±0.33b	906.9±5.7b
D4	1.52±0.02d	16.63±0.17a	3.84±0.02a	804±11b	31.35±0.24c	32.11±0.37b	787.0±5.6c
Comparison of densities	*P* < 0.01	*P* > 0.05	*P* > 0.05	*P* < 0.05	*P* < 0.05	*P* < 0.05	*P* < 0.05
Standard error difference	0.030	0.385	0.107	14.05	0.417	0.425	29.89

SP, number of spikes per plant; FSN, number of fertile spikelets per spike; SSN, number of sterile spikelets per spike; SN, number of spikes m^-2^; GN, number of grains per spike; TGW, 1,000-grain weight; GY, grain yield m^-2^; D1, 75 plants m^-2^; D2, 225 plants m^-2^; D3, 375 plants m^-2^; D4, 525 plants m^-2^. Data are means of three replicates. Standard error difference represents standard error of the difference between means. Means within each cultivar followed by a different letter are significantly different at *P* < 0.05.

The FSN, GN, and spike weight (SW) of Wennong6 were higher than those of Jimai20 under the same plant density conditions (Table 3). The seed-setting traits of the main stem spike were superior to those of the tiller spike in both cultivars, and basal SSN was significantly higher than top SSN. Significant decrease was observed in SW of both cultivars with the increasing plant density except SW in main stem of Jimai20. (Table 3).

**Table 3:** Seed-setting traits of main stem and tiller spikes in two wheat cultivars, ‘Wennong6’ and ‘Jimai20’, grown under four different plant densities.

**Treatment**	**Main stem spike**				
	**FSN**	**Top SSN**	**Basal SSN**	**GN**	**SW (g)**
Wennong6					
D1	20.81±0.25a	0.61±0.01a	2.17±0.14a	61.80±1.02a	2.53±0.01a
D2	20.61±0.11ab	0.63±0.01a	2.32±0.10a	60.68±0.89a	2.45±0.02b
D3	20.39±0.17ab	0.53±0.01b	2.27±0.06a	53.83±1.14b	2.23±0.02c
D4	20.01±0.06b	0.39±0.01c	2.24±0.06a	50.14±0.11c	2.20±0.02c
Jimai20					
D1	18.73±0.18a	0.47±0.05a	2.55±0.06b	41.05±0.46a	1.94±0.02a
D2	18.68±0.14a	0.54±0.02a	2.84±0.01a	38.02±0.66b	1.85±0.01a
D3	18.14±0.04b	0.53±0.02a	2.83±0.04a	33.01±1.05c	1.79±0.03a
D4	16.97±0.13c	0.37±0.01b	2.99±0.04a	32.41±1.00c	1.79±0.00a
Comparison of densities	*P* < 0.05	*P* < 0.05	*P* > 0.05	*P* < 0.05	*P* < 0.05
Standard error difference	0.308	0.03	0.11	1.24	0.028
**Treatment**	**Tiller spike**				
	**FSN**	**Top SSN**	**Basal SSN**	**GN**	**SW (g)**
Wennong6					
D1	18.83±0.07a	1.47±0.06b	2.91±0.04b	51.83±0.67a	2.11±0.06a
D2	18.29±0.13b	1.45±0.03b	2.77±0.03c	51.08±0.46ab	2.00±0.01ab
D3	18.24±0.09b	2.13±0.08a	3.65±0.03a	48.37±1.00bc	1.93±0.02b
D4	17.69±0.16c	2.14±0.03a	3.62±0.02a	47.38±1.11c	1.93±0.07b
Jimai20					
D1	16.26±0.07a	0.47±0.03b	2.82±0.06c	39.02±0.12a	1.65±0.02a
D2	16.38±0.21a	0.70±0.01a	3.20±0.03b	36.05±0.50bc	1.57±0.02b
D3	16.24±0.24a	0.69±0.03a	3.84±0.03a	31.63±0.14c	1.44±0.03c
D4	15.56±0.43b	0.46±0.02a	3.82±0.05a	30.20±0.21d	1.39±0.01c
Comparison of densities	*P* < 0.05	*P* < 0.05	*P* < 0.05	*P* < 0.05	*P* < 0.05
Standard error difference	0.300	0.058	0.058	0.905	0.05

FSN, number of fertile spikelets per spike; SSN, number of sterile spikelets per spike; GN, number of grains per spike; SW, spike weight; D1, 75 plants m^-2^; D2, 225 plants m^-2^; D3, 375 plants m^-2^; D4, 525 plants m^-2^. Data are means of three replicates. Standard error difference represents standard error of the difference between means. Means within each cultivar followed by a different letter are significantly different at *P* < 0.05.

### Effects of plant density on the grain number per spikelet at different spikelet positions

**Fig 2:**
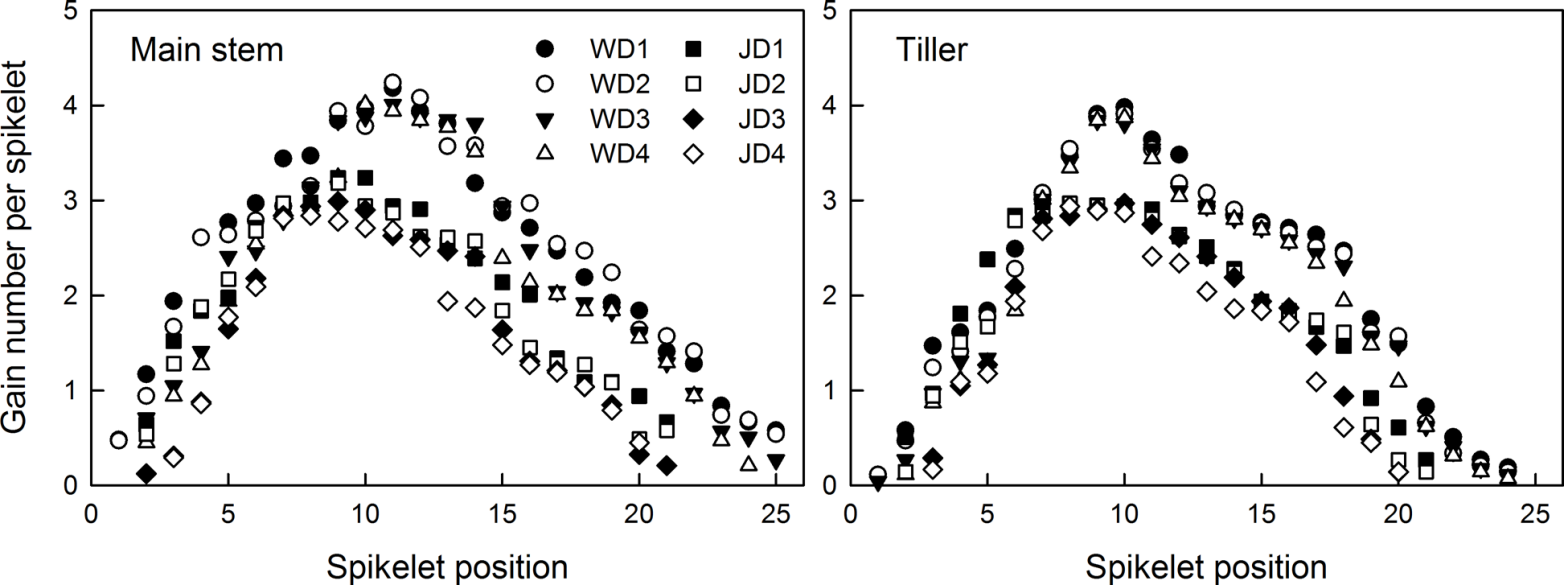
Grain number per spikelet at different spikelet positions in two wheat cultivars ‘Wennong6’ (W) and ‘Jimai20’ (J), grown under four different plant densities. D1, 75 plants m^-2^; D2, 225 plants m^-2^; D3, 375 plants m^-2^; D4, 525 plants m^-2^.

In the main stem and tiller spikes, grain number per spikelet (GNS) first increased and then decreased from the bottom to the top spikelets, showing a parabolic change. The GNS of the main stem spike was obviously higher than that of the tiller spike (Fig 2). The correlation between the grain number per spikelet and its position in the main stem and tiller spike were significant (Table 4). However, the spatial distribution of GNS was not symmetrical. The highest grain number was observed in the 7^th^-13^th^ spikelets of Wennong6 and the 8^th^-11^th^ spikelets of Jimai20, followed by the 3^rd^-6^th^ and 3^rd^-7^th^ spikelets and the 14^th^-20^th^ and 12^th^-18^th^ spikelets of Wennong6 and Jimai20, respectively. Therefore, the highest grain number was observed in the middle spikelets, followed by the basal, upper, and top spikelets. GNS increased from the basal to the middle spikelets, following a linear trend, and then reduced slowly from the middle to the top spikelets. The differences between the grain numbers at different spikelet positions of the basal spikelets were much higher than those of the middle spikelets.

**Table 4:** Fitting equations of grain number per spikelet (y) at different spikelet positions (x) in two wheat cultivars, ‘Wennong6’ and ‘Jimai20’, grown under four different plant densities.

Treatment	Main stem spike		Tiller spike	
	Equation	Coefficient (*r*)	Equation	Coefficient (*r*)
Wennong6				
D1	y = -0.0217x^2^ + 0.5113x + 0.5542	0.874**	y = -0.0265x^2^ + 0.6257x - 0.2957	0.917**
D2	y = -0.0220x^2^ + 0.5303x + 0.4251	0.897**	y = -0.0268x^2^ + 0.6354x - 0.4006	0.913**
D3	y = -0.0245x^2^ + 0.5995x - 0.2195	0.809**	y = -0.0270x^2^ + 0.6500x - 0.6641	0.898**
D4	y = -0.0264x^2^ + 0.6030x - 0.3884	0.871**	y = -0.0274x^2^ + 0.6570x - 0.7638	0.886**
Jimai20				
D1	y = -0.0271x^2^ + 0.5783x - 0.1306	0.891**	y = -0.0280x^2^ + 0.5958x - 0.2601	0.917**
D2	y = -0.0272x^2^ + 0.5781x - 0.1494	0.884**	y = -0.0297x^2^ + 0.6384x - 0.5884	0.923**
D3	y = -0.0306x^2^ + 0.6093x - 0.3324	0.872**	y = -0.0315x^2^ + 0.6874x - 1.0190	0.930**
D4	y = -0.0279x^2^ + 0.6033x - 0.7403	0.861**	y = -0.0295x^2^ + 0.6354x - 0.8985	0.884**

**Significant at *P* < 0.01.

D1, 75 plants m^-2^; D2, 225 plants m^-2^; D3, 375 plants m^-2^; D4, 525 plants m^-2^.

The GNS of both cultivars tended to decline with the increasing plant density (Fig 2). The grain number per spikelet at the basal (1^st^ and 2^nd^) and top spikelet positions was lower than that at other positions. The grain number at the basal spikelet positions (except for the 1^st^ and 2^nd^) reduced significantly with the increasing plant density, while that at the middle and upper spikelet positions showed no significant change. The results indicated that plant density had a stronger effect on the seed-setting traits at the basal spikelet positions than those of other spikelet positions.

### Spatial distribution of single-grain weight at different spikelet positions

**Fig 3:**
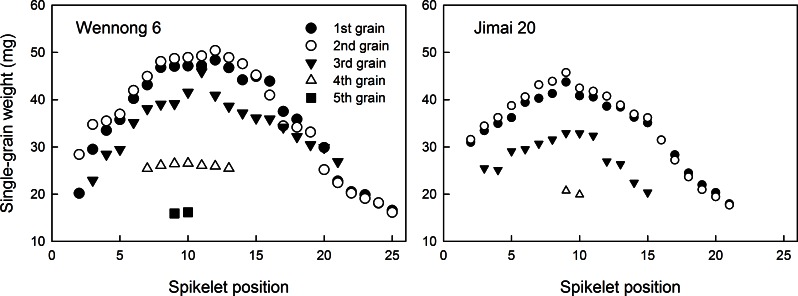
Distribution of single-grain weight at different spikelet positions in two wheat cultivars, ‘Wennong6’ (W) and ‘Jimai20’ (J), grown under four different plant densities. D1, 75 plants m^-2^; D2, 225 plants m^-2^; D3, 375 plants m^-2^; D4, 525 plants m^-2^.

The effects of spikelet and grain positions on single-grain weight (SGW) varied with the grain number per spikelet (Fig 3). SGW was significantly positively correlated with GW and grain position and showed a parabolic change at the 1^st^, 2^nd^, and 3^rd^ grain positions (Table 5). The SGW at the 1^st^ grain position was similar to that at the 2^nd^ grain position, but higher than those at the 3^rd^ and 4^th^ grain positions. In addition, the SGW of the basal and middle spikelets was the highest at the 2^nd^ grain position. At the 1^st^ and 2^nd^ grain positions, the SGW of middle spikelets (7th–15th spikelet) of Wennong6 was heavier (p<0.01) than that of Jimai20 (Fig 3).

**Table 5:** Fitting equations of single-grain weight (y, mg) for a specific grain position at different spikelet positions (x) in two wheat cultivars, ‘Wennong6’ and ‘Jimai20’.

Grain position	Equation	Coefficient (*r*)
Wennong6		
1^st^	y = -0.2124x^2^ + 5.0286x + 16.530	0.927**
2^nd^	y = -0.1946x^2^ + 4.2832x + 23.358	0.912**
3^rd^	y = -0.1955x^2^ + 4.6533x + 13.027	0.863**
4^th^	y = -0.1119x^2^ + 2.2167x + 15.453	0.924**
Jimai20		
1^st^	y = -0.1841x^2^ + 3.0321x + 28.600	0.964**
2^nd^	y = -0.2019x^2^ + 3.6623x + 25.880	0.961**
3^rd^	y = -0.2845x^2^ + 4.8014x + 11.961	0.911**

**Significant at *P* < 0.01.

### Effects of plant density on the spatial distribution of grain weight per spikelet

**Fig 4:**
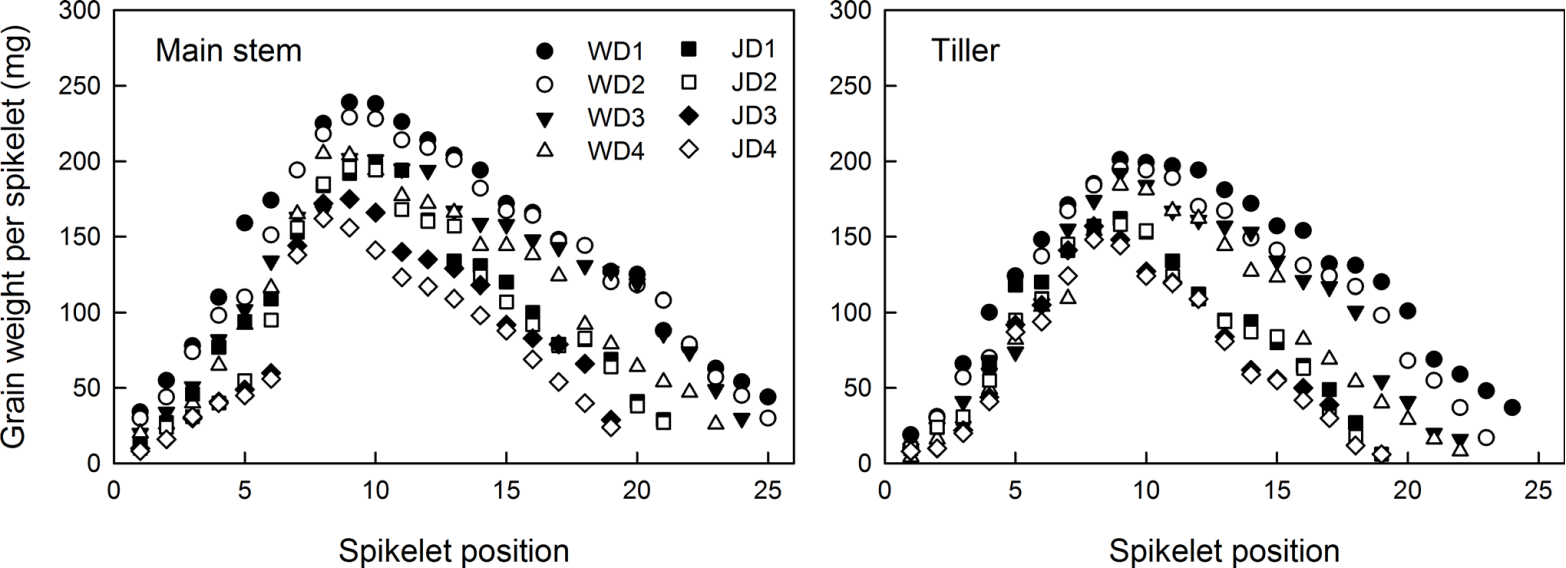
Distribution of grain weight per spikelet at different spikelet positions in two wheat cultivars, ‘Wennong6’ (W) and ‘Jimai20’ (J), grown under four different plant densities. D1, 75 plants m^-2^; D2, 225 plants m^-2^; D3, 375 plants m^-2^; D4, 525 plants m^-2^.

The grain weight per spikelet (GWS) of the main stem and tiller spikes showed parabolic changes, since it first increased and then decreased from the bottom to the top (Fig 4). The GWS at the middle spikelet position (8^th^–12^th^) was the highest, while the basal spikelets had higher differences in GWS that the middle or top spikelets. In addition, the GWS of the main stem spike was much higher than that of the tiller spike. The results also showed that GWS was significantly correlated with the spikelet position in the main stem and tiller spikes (Table 6).

**Table 6:** Fitting equations of grain weight per spikelet (y, mg) at different spikelet positions (x) in two wheat cultivars, ‘Wennong6’ and ‘Jimai20’, grown under four different plant densities.

Treatment	Main stem spike		Tiller spike	
	Equation	Coefficient (*r*)	Equation	Coefficient (*r)*
Wennong6				
D1	y = -1.2629x^2^ + 30.709x + 21.963	0.869**	y = -1.3017x^2^ + 31.636x - 4.5899	0.931**
D2	y = -1.2817x^2^ + 31.761x + 4.8035	0.903**	y = -1.4388x^2^ + 33.874x - 22.988	0.924**
D3	y = -1.2645x^2^ + 31.577x - 14.210	0.928**	y = -1.5326x^2^ + 35.518x - 38.896	0.921**
D4	y = -1.4182x^2^ + 33.439x - 23.604	0.866**	y = -1.4917x^2^ + 34.696x - 45.630	0.917**
Jimai20				
D1	y = -1.6372x^2^ + 35.936x - 30.931	0.873**	y = -1.7869x^2^ + 34.968x - 30.907	0.903**
D2	y = -1.6657x^2^ + 37.386x - 48.280	0.822**	y = -1.7828x^2^ + 34.882x - 35.109	0.895**
D3	y = -1.7275x^2^ + 37.109x - 55.257	0.813**	y = -1.7040x^2^ + 33.213x - 37.348	0.844**
D4	y = -1.6053x^2^ + 33.587x - 47.445	0.811**	y = -1.6420x^2^ + 32.037x - 37.803	0.854**

**Significant at *P* < 0.01.

D1, 75 plants m^-2^; D2, 225 plants m^-2^; D3, 375 plants m^-2^; D4, 525 plants m^-2^.

The GWS of the main stem and tiller spikes of Wennong6 was higher than that of Jimai20, especially under low plant density conditions (Fig 4). GWS tended to decline with the increasing plant density (Fig 4), while it was lower at the basal (1^st^ and 2^nd^) and top spikelet positions compared with that at the middle spikelet positions. The GWS at the basal spikelet position (except for 1^st^ and 2^nd^) significantly reduced with the increasing plant density. However, the GWS at the middle and upper spikelet positions showed no significant changes under different plant density conditions. Plant density had a stronger effect on the GWS of Wennong6 than that of Jimai20.

### Effects of plant density on the distribution of single-grain weight

**Fig 5:**
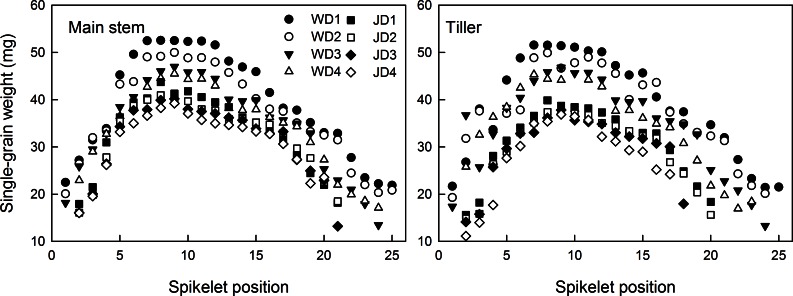
Distribution of single-grain weight at different spikelet positions in two wheat cultivars, ‘Wennong6’ (W) and ‘Jimai20’ (J), grown under four different plant densities. D1, 75 plants m^-2^; D2, 225 plants m^-2^; D3, 375 plants m^-2^; D4, 525 plants m^-2^.

The SGW of the main stem and tiller spikes showed parabolic changes, since it first increased and then decreased from the bottom to the top (Fig 5). SGW was the lowest at the basal (1^st^ and 2^nd^) and top spikelet positions. In Wenong6, the SGW in the middle spikelets of the main stem spike did not differ significantly from that of the tiller spike, whereas in Jimai20, the SGW in the middle spikelets of the main stem spike was higher than that of the tiller spike. SGW was significantly correlated with the grain position (Table 7). However, the spikelet position had a stronger effect on the SGW of the main stem spike than that of the tiller spike. The SGW of Wennong6, especially at the middle and upper spikelets, significantly reduced with increasing plant density and varied more greatly in the main stem spike than in the tiller spike. However, plant density had a weak effect on the SGW of Jimai20.

**Table 7:** Fitting equations of single-grain weight (y, mg) at different spikelet positions (x) in two wheat cultivars, ‘Wennong6’ and ‘Jimai20’, grown under four different plant densities.

Treatment	Main stem spike		Tiller spike	
	Equation	Coefficient (*r*)	Equation	Coefficient (*r*)
Wennong6				
D1	y = -0.2004x^2^ + 4.6732x + 22.668	0.861**	y = -0.1886x^2^ + 4.3293x + 24.021	0.858**
D2	y = -0.1832x^2^ + 4.2374x + 22.091	0.865**	y = -0.2145x^2^ + 4.9155x + 18.987	0.878**
D3	y = -0.2164x^2^ + 4.8388x + 17.671	0.954**	y = -0.2062x^2^ + 4.5155x + 19.723	0.919**
D4	y = -0.2272x^2^ + 5.4229x + 11.069	0.815**	y = -0.2435x^2^ + 5.4215x + 13.183	0.817**
Jimai20				
D1	y = -0.2530x^2^ + 5.4961x + 11.732	0.910**	y = -0.3069x^2^ + 5.9571x + 10.751	0.924**
D2	y = -0.2388x^2^ + 5.3670x + 10.169	0.876**	y = -0.2755x^2^ + 6.0210x + 6.7899	0.949**
D3	y = -0.2511x^2^ + 5.5623x + 8.878	0.906**	y = -03937x^2^ + 8.1616x + 3.7644	0.868**
D4	y = -0.2757x^2^ + 5.9607x + 6.647	0.854**	y = -0.4496x^2^ + 9.0132x - 7.9437	0.925**

**Significant at *P* < 0.01.

D1, 75 plants m^-2^; D2, 225 plants m^-2^; D3, 375 plants m^-2^; D4, 525 plants m^-2^.

### Relationship among grain weight per spikelet, grain number, and single-grain weight

**Fig 6:**
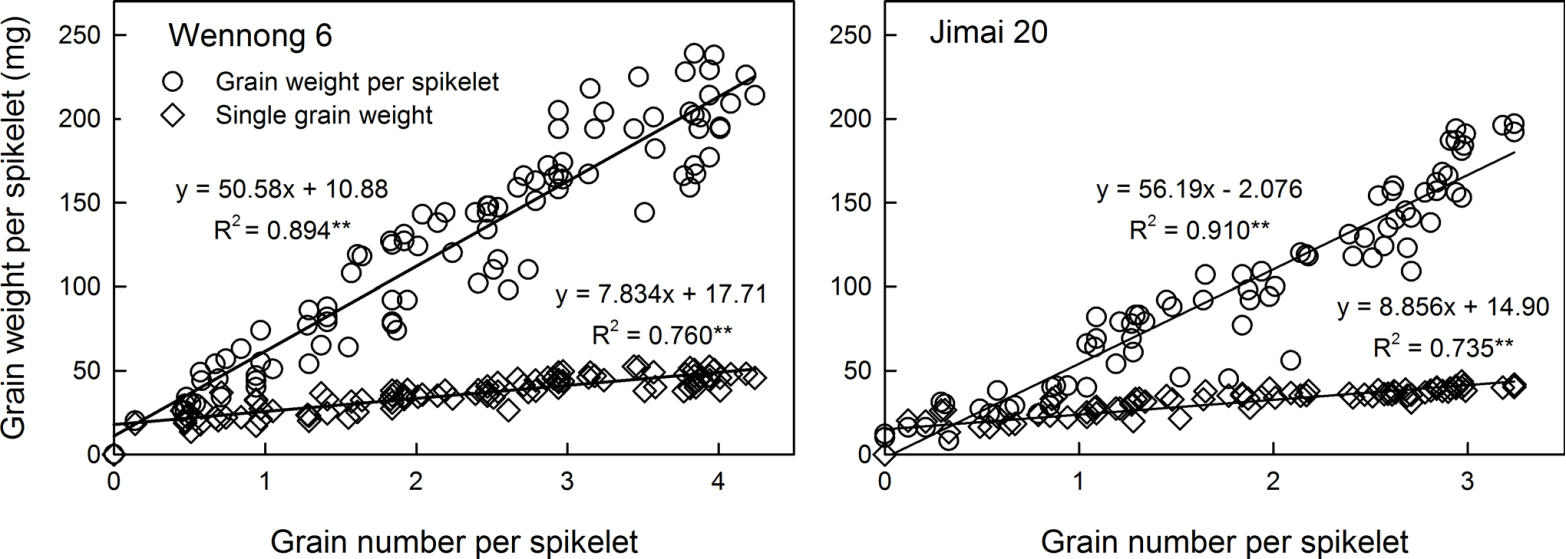
Relationships between grain weight per spikelet, grain number, and single-grain weight at different spikelet positions in two wheat cultivars, ‘Wennong6’ and ‘Jimai20’.

GWS was significantly positively correlated with GNS (Fig 6) and the latter with SGW. In addition, GWS was significantly positively correlated with SGW, and the correlation coefficients were 0.855 and 0.671 for Wennong6 and Jimai20, respectively. Both GNS and GN had a strong positive effect on GWS.

### Distribution of single-grain weight at different grain positions

**Fig 7:**
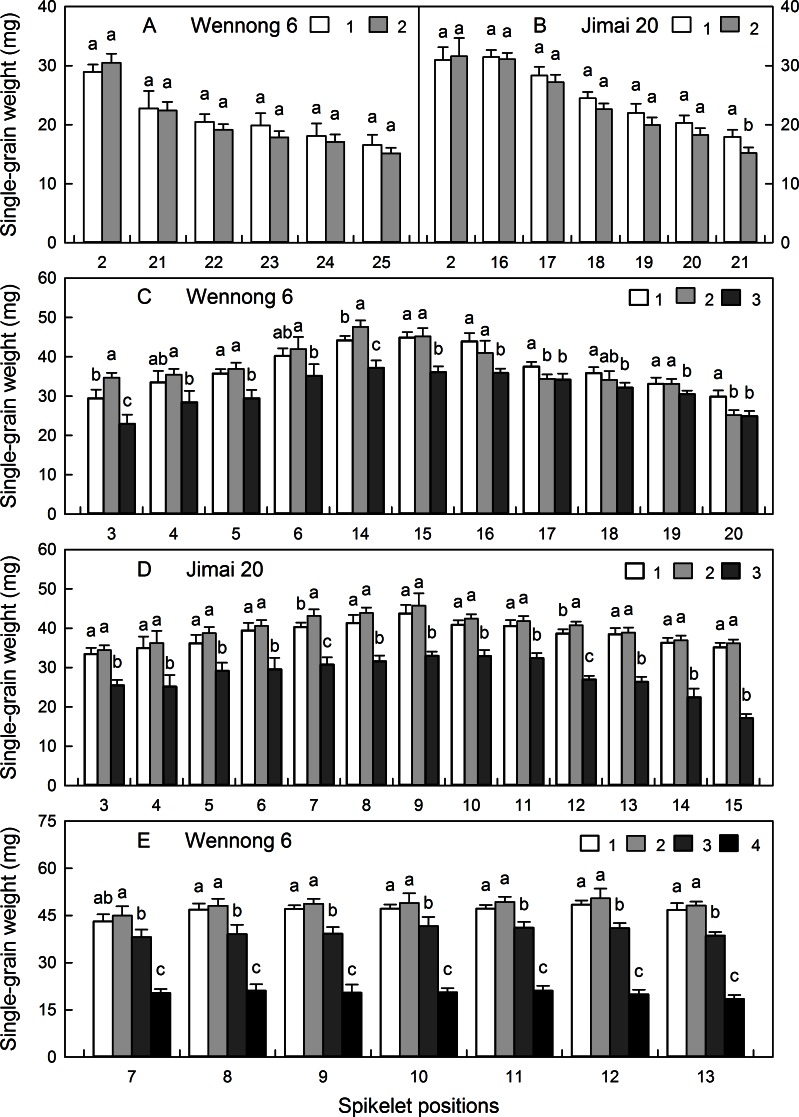
Single-grain weight of two-grain (A and B), three-grain (C and D), and four-grain (E) spikelets at different grain positions in two wheat cultivars, ‘Wennong6’ and ‘Jimai20’.

The effects of spikelet and grain positions on SGW varied with the GN of spikelets and cultivars. The two-grain spikelets were mainly distributed at the basal (2^nd^) and top spikelet positions. In the basal two-grain spikelets of both cultivars, the SGW at the 2^nd^ grain position was higher than that at the 1^st^ grain position, whereas the opposite occurred in the top two-grain spikelets (Fig 7A and 7B). In the three-grain spikelets, the distribution of SGW was different between cultivars. In the basal (3^rd^–6^th^) and middle (14^th^ and 15^th^) three-grain spikelets of Wennong6, SGW was the highest at the 2^nd^ grain position, followed by the 1^st^ and 3^rd^ grain positions, while in the top spikelets, SGW was the highest at the 1^st^ grain position, followed by the 2^nd^ and 3^rd^ grain positions (Fig 7C). In the basal and middle three-grain spikelets of Jimai20, SGW was the highest at the 2^nd^ grain position, followed by the 1^st^ and 3^rd^ grain positions (Fig 7D). In the four- and five-grain spikelets of Wennong6, SGW was the highest at the 2^nd^ grain position, followed by the 1^st^, 3^rd^, 4^th^, and 5^th^ grain positions (Fig 7E). Regardless the spikelet and grain positions, SGW was the highest at the 1^st^ and 2^nd^ grain positions and the lowest at the 3^rd^ and 4^th^ grain positions.

## Discussion

The variation of GN and grain weight (GW) was a major factor for determining the effects of spikelet and grain positions on the grain set and matter accumulation [9, 11, 12]. Li et al. [37] reported that GWS, GNS, and SGW at different spikelet and grain positions, and the seed-setting rate showed a partial spindle distribution and differed between the main stem and tiller spikes. Our results showed that GN, GWS, and GW at different spikelet positions from the bottom to the top showed parabolic changes, which were in agreement with previous studies [9, 11, 12]. It has been suggested that GN depended on the fecundity of basic spikelets, while GW and GY depended on matter accumulation in the grains of top spikelets and was mainly associated with the development of spikelets and florets [38].

It has been reported that the degree and rate of grain number and grain weight highly differ between spikelets based on their positions in the wheat spike [11, 12, 29]. Large differences have been observed between florets in the amount and size of vascular bundles, carbon amount, sink capacity, endogenous hormone levels, activity and/or gene expression of enzymes involved in sucrose-to-starch conversion, and assimilate transportation, resulting in an asynchronous grain development and nutriment accumulation as well as in yield differences among spikelets and grains [13–16, 18–21, 23]. Jiang et al. [39] suggested that superior grains have higher sucrose contents at the early and middle grain filling, and thus, a greater ability for starch synthesis. Ru et al. [40] found that TGW in wheat was different at different spikelet and grain positions. In this study, we found that the effects of spikelet and grain positions on SGW varied with the grain number of spikelet. The SGW at the 1^st^ grain position was similar to that at the 2^nd^ grain position and higher than that at the 3^rd^ and 4^th^ grain positions. In the basal (2^nd^) to top (15^th^) two-grain, three-grain, and four-grain spikelets, the SGW at the 2^nd^ grain position was higher compared to that at the 1^st^ grain position. These findings were in agreement with those reported by Qu et al. [29] and Pan et al. [11], but in disagreement with those of Zhu [41], probably due to differences in the used wheat varieties. The variation in the 2^nd^ grain position was a major factor for determining the effects of spikelet and grain positions on grain development, the spatial distribution of grain weight, and matter accumulation under different conditions as well as a major target for improving yield [9]. The results suggested that wheat production could be boosted by enhancing the GW at the 3^rd^ and 4^th^ grain positions, while maintaining it at the 1^st^ and 2^nd^ grain positions. Wennong6 has a greater potential in improving yield than Jimai20, since the former has a higher GW at the 3^rd^ and 4^th^ grain positions than the latter.

Plant density is an important factor that influences the growth and yield in wheat [13, 24, 25]. In this study, GN and GW reduced with the increasing plant density. Li et al. [37] reported that under dense planting, the grain number at the 3^rd^ and 4^th^ grain positions and the 1^st^ and 2^nd^ grain positions of the basal and top spikelets decreased. In this study, we found that the seed-setting characteristics and GW of Wennong6 were superior to those of Jimai20. Meanwhile, the seed-setting traits of the main stem spike were superior to those of the tiller spike. SW showed decreasing trends with the increasing plant density, and the decrease was even greater in Wennong6. Therefore, plant density probably had a stronger effect on cultivars with higher grain numbers, suggesting that the interaction of genotype and plant density may determine the variation in the grain number and matter accumulation of the wheat spike.

Previous studies showed that the differences in SW under different plant density conditions were significant in wheat [9, 42] and rice [43]. A very high plant density increases SN, but decreases GWS in wheat [29]; therefore, extreme plant density conditions may have a negative effect on yield. The wheat cultivar Jimai20 is a medium-spike cultivar, which produces more tillers per grain than the larger spike cultivar at the same density. As the density increases, tillers produced by a single grain become less. There is considerable compensation by the crops grown at low densities, which was in agreement with Whaley et al. [44]. SN is determined by total tillers, which is equal to the product of density and tillers produced by a single grain. Thus, a targeted SN might be shaped from different density due to genotypes. The differences between genotypes are reflected in spikelet positions and form the substantive characteristics of spikes between cultivars [45]. Therefore, in wheat production, we should properly trade off the relationship between GN and GW, and then improve these two yield components along with SW in order to reach their maximum potential.

## Conclusions

FSN, GN, and SGW significantly decreased with the increasing plant density. The seed-setting characteristics in the main stem spike were relatively stable, while plant density affected the yield by controlling the seed-setting characteristics in the tiller spike. To boost wheat production, we should choose the appropriate plant density based on cultivars to maintain the seed-setting characteristics of the tiller spike, increase the seed-setting rate by supplying sufficient nutrients, mainly nitrogen nutrient, to decrease the number of basal and top sterile spikelets, and enhance the grain weight at the 3^rd^ and 4^th^ grain positions, while maintaining it at the 1^st^ and 2^nd^ grain positions.

## Supporting Information

S1 Dataset Figs SN, SGW and GWS at Different Spikelet and Grain Positions. Figs 2–7 were plotted based on this excel file.
(XLSX)

S2 Dataset Tables. Seed-setting traits of spike, yield components, GNS, SGW and SW at Different Spikelet and Grain Positions. Tables 2–7 were created based on this excel file.
(XLSX)
